# Evaluation of novel metrics derived from a brief smartphone cognitive task for predicting disease severity in frontotemporal dementia

**DOI:** 10.1002/alz.093502

**Published:** 2025-01-03

**Authors:** Jack C. Taylor

**Affiliations:** ^1^ Memory and Aging Center, UCSF Weill Institute for Neurosciences, University of California, San Francisco, San Francisco, CA USA

## Abstract

**Background:**

Sensitive assessments capable of detecting cognitive changes are important for improving early identification of symptoms in frontotemporal dementia (FTD). The ALLFTD Mobile App features smartphone cognitive tests that capture a range of FTD symptoms. We have previously shown that the flanker task from this battery is reliable and capable of detecting early changes in FTD. Here we generate features from the digital flanker task and utilize machine learning algorithms to test their incremental utility for detecting cognitive changes in FTD.

**Method:**

Sample included 50 symptomatic and 70 prodromal, sporadic and familial FTD participants (CDR®+NACC‐FTLD = 1 or 0.5, respectively) with a range of FTD syndromes, and 70 controls (CDR®+NACC‐FTLD = 0, no pathogenic mutation) from the ALLFTD Consortium. Participants completed a two‐minute, 100‐trial digital flanker assessment on the ALLFTD Mobile App. We extracted 301 distribution metrics, based on reaction time and trial type, and 15 traditional metrics using an in‐house pipeline. Random forest models were trained using 5‐fold cross validation to classify controls from prodromal or from symptomatic individuals. The top 10 metrics of importance based on SHAP value were then entered as predictors in a standard logistic regression model and compared to a logistic model including only total score (speed + accuracy) as the predictor.

**Result:**

In all comparisons, logistic regressions achieved higher classification accuracies than the random forest models. When using random forest to classify controls vs prodromal and controls vs symptomatic, AUCs were 0.69 and 0.81, respectively. Logistic model AUCs for classifying controls vs prodromal were 0.85 using total score alone and 0.81 using all top 10 metrics. When classifying controls vs symptomatic, model AUCs were 0.84 using total score alone and 0.91 using all top 10 metrics.

**Conclusion:**

We tested the use of experimental features derived from a brief two‐minute test to distinguish healthy individuals from prodromal or symptomatic FTD. Classification accuracies were moderate to high, suggesting that while traditional measures continue to perform well, novel features provide an improvement in accuracy. Future directions include analogous investigations of other smartphone cognitive tasks collected through this battery.

